# The Role of Toll-Like Receptor 9 in Chronic Stress-Induced Apoptosis in Macrophage

**DOI:** 10.1371/journal.pone.0123447

**Published:** 2015-04-17

**Authors:** Yanxiao Xiang, Hui Yan, Jun Zhou, Qi Zhang, Gregory Hanley, Yi Caudle, Gene LeSage, Xiumei Zhang, Deling Yin

**Affiliations:** 1 Department of Internal Medicine, College of Medicine, East Tennessee State University, Johnson City, Tennessee 37614, United States of America; 2 Department of Pharmacology, Shandong University School of Medicine, Jinan, People's Republic of China; 3 Department of Radiology, Shuguang Hospital, Shanghai University of Traditional Chinese Medicine, Shanghai, People's Republic of China; 4 Laboratory Animal Resources, College of Medicine, East Tennessee State University, Johnson City, Tennessee 37614, United States of America; University of São Paulo, BRAZIL

## Abstract

Emerging evidence implied that chronic stress has been exerting detrimental impact on immune system functions in both humans and animals. Toll-like receptors (TLRs) have been shown to play an essential role in modulating immune responses and cell survival. We have recently shown that TLR9 deficiency protects against lymphocyte apoptosis induced by chronic stress. However, the exact role of TLR9 in stress-mediated change of macrophage function remains unclear. The results of the current study showed that when BALB/c mice were treated with restraint stress (12 h daily for 2 days), the number of macrophages recruited to the peritoneal cavity was obviously increased. Results also demonstrated that the sustained effects of stress elevated cytokine IL-1β, TNF-α and IL-10 production yet diminished IFN-γ production from macrophage, which led to apoptotic cell death. However, TLR9 deficiency prevented the chronic stress-mediated accumulation of macrophages. In addition, knocking out TLR9 significantly abolished the chronic stress-induced imbalance of cytokine levels and apoptosis in macrophage. TLR9 deficiency was also found to reverse elevation of plasma IL-1β, IL-10 and IL-17 levels and decrease of plasma IFN-γ level under the condition of chronic stress. These results indicated that TLR9-mediated macrophage responses were required for chronic stress-induced immunosuppression. Further exploration showed that TLR9 deficiency prevented the increment of p38 MAPK phosphorylation and reduction of Akt/Gsk-3β phosphorylation; TLR9 deficiency also attenuated the release of mitochondrial cytochrome c into cytoplasm, caused upregulation of Bcl-2/Bax protein ratio, downregulation of cleavage of caspase-3 and PARP, as well as decreased TUNEL-positive cells in macrophage of stressed mice. Collectively, our studies demonstrated that deficiency of TLR9 maintained macrophage function by modulating macrophage accumulation and attenuating macrophage apoptosis, thus preventing immunosuppression in restraint-stressed mice.

## Introduction

Experimental studies and clinical observations have indicated that stress serves as an important risk factor in the etiology of infectious and autoimmune diseases [1, 2]. Both acute and chronic stress has been found to have dramatic impacts on the immunological parameters in both humans and animals. Data suggested a positive effect of acute stress on the immune system, while chronic stress frequently leads to immunosuppression [3]. These effects at least partly depend on the function of and apoptotic cell death of immune cells. Numerous studies have revealed that chronic stress leads to a decrease of thymocytes and splenocytes by a mechanism associated with stress-induced lymphocyte apoptosis [4, 5]. As one of the most important immune cells, macrophages might express inducible nitric oxide synthase (iNOS), H_2_O_2_, tumor necrosis factor (TNF)-α and interleukin (IL)-1, which participate in the macrophage-induced suppression of immune responses[6] [7]. However, it remains unknown whether macrophages are involved with the immune suppression due to chronic stress. Recent study reveals that stressful life events are associated with altered levels of macrophages in rat models of prostate and breast cancers[8], we hypothesize that chronic stress plays immunosuppressive function partially by inducing macrophage responses. Here, we employed the physical restraint stress mouse model to examine the relationship between chronic stress and macrophage, and to explore the effect of chronic stress on macrophage apoptosis and the possible molecular mechanism.

Macrophages play important roles in regulating immunity by virtue of their ability to secrete a multitude of proinflammatory cytokines and chemokines. Many studies have shown that toll-like receptors (TLRs) modulate the activation of macrophages by pathogens. Among the subsets of TLRs, several pattern recognition receptors have previously been implicated in the chronic stress-induced immune response, including TLR2 and TLR4, as well as the downstream the phosphoinositide 3-kinase (PI3K)/Akt signaling [5, 9]. Previous studies have indicated an involvement of TLR9 in the development of innate immune responses, but the precise role of TLR9 and the underlying mechanisms in the macrophage response after chronic stress exposure is still poorly documented. Recent studies reported that stressed mice showed increased intestinal permeability which resulted in bacterial translocation to the peritoneal cavity [10]. These peritoneal bacteria are a major source of CpG DNA, which can trigger the activation of macrophage TLR9 and cause immune response[11]. Recent studies from us and others have revealed that activation of TLR9 signaling triggers activation of pro-apoptotic signaling pathways, and cause cell apoptosis in various system [11–13].

TLR9 stimulation activates PI3K/Akt and mitogen-activated protein kinase (MAPK) signaling pathway[14]. Akt is an important cellular factor which exerts critical roles in regulating many cellular functions, such as cellular activation, inflammatory response, and apoptosis[15]. GSK-3β is a constitutively active enzyme that is inactivated by Akt which regulates cell survival and apoptosis [16]. MAPKs are associated with some important aspects of immune responses [17]. Among MAPK families, p38 MAPK is easily activated by stress signals [18]. Earlier studies established that activation of p38 MAPK and down-regulation of Akt kinase led to leucocytes apoptosis by the disruption of Bcl-2, caspase activation and subsequent apoptotic features[19].

A distinctive feature of activated macrophages is their capacity to rapidly generate TNF-α in response to diverse stimuli. In addition to producing TNF-α, activated macrophages secrete the cytokine IL-10 which contributes to the down-regulation of IFN-γand consequently, in the apoptosis process of macrophage, highlighting the importance of macrophage in innate and adaptive immune responses[20]. This report investigates mechanisms by which TLR9 inhibition suppresses chronic stress-induced imbalance of cytokines production. We demonstrated that TNF-α, IL-1βand IL-10 production, as well as p38 activation, cleaved caspase-3 and cleaved poly ADP-ribose polymerase (PARP) induced by chronic stress were impaired in macrophages from TLR9-deficient mice. We also showed that TLR9 deficiency did restore chronic stress-impaired IFN-γproduction, Akt/GSK-3β phosphorylation and Bcl-2/Bax ratio in macrophage.

## Materials and Methods

### Experimental animals

Breeding pairs of TLR9 knockout (not a functional knockout) mice on a BALB/c background were kindly provided by Dr. Shizuo Akira (Osaka University, Osaka, Japan) via Dr. Dennis Klinman (National Cancer Institute, Frederick, MD). Wild type BALB/c male mice were purchased from the Harlan (Indianapolis, Indiana) and all mice were kept in the Division of Laboratory Animal Resources at East Tennessee State University (ETSU), a facility accredited by the Association for the Assessment and Accreditation of Laboratory Animal Care (AAALAC). All animals were maintained in a specific pathogen-free room under controlled conditions at the room temperature (23±1℃) with a 12-h light-dark cycle. All experiments were adhered to the animal use protocol approved by the ETSU Committee on Animal Care.

### Experimental model of restraint stress

All mice (male, weight 23~25g) were healthy and six to eight-week-old. The protocol used to establish chronic physical restraint model was proved to be effective in our laboratory as well as others [21]. Briefly, wild type mice and TLR knockout mice were randomly divided into 2 groups, 7 in each group, respectively. Each individual mouse of stress group was placed in a 50-ml polypropylene conical centrifuge tube (Corning, NY). The tubes were arranged with multiple punctures for ventilation. Mice were restricted horizontally in the tubes for 12 h (from21:00 to next day 9:00) followed by a 12 h rest (from 9:00 to 21:00). The stressed mice were kept next to each other. The stressed mice were provided with food and water during the rest period in an ordinary cage. Food and water were provided to control littermates in their original cage only during the 12 h rest. The cages were transparent, well-ventilated and only contained food, water and bedding materials during the rest period. Observations in our laboratory showed that during restraint, the mice did not suffer from any physical suppression or pain. After 2 cycles, mice were humanely killed by cervical dislocation for the subsequent experiments.

### Isolation of peritoneal macrophages

After the two cycles of stress finished, mice were humanely killed by cervical dislocation, and peritoneal macrophages were collecting by injecting 5 ml of phosphate-buffered saline (PBS) into the peritoneal cavity. The cell suspension was cultured with RPMI-1640 containing 10% fetal bovine serum (FBS) for 60 min at 37°C in to allow the macrophages to adhere as described by Mantovani [22]. After being washed in PBS, the non-adherent cells were removed. The purity of macrophages was >95%.

### Determination of apoptosis by TUNEL assay

TUNEL assay was performed according to our previous study [23]. Apoptotic nuclear DNA fragments were investigated using the In Situ Cell Death Detection kit (Roche Diagnostic, Indianapolis, IN). Briefly, macrophages (5 × 10^5^ cells) from wild type and TLR9 knockout mice were fixed in 4% formaldehyde/PBS for 20 min at 37°C，permeabilized in 0.1% sodium citrate solution containing 0.1% Triton X-100, for 10 min, after that, the sections were incubated with 50 μL of TUNEL reaction mixture for 60 min at 37°C. After convert-AP incubation, 50 μL of substrate solution was placed on the slices. Finally, sections were conterstained with haematoxylin. Slices were observed under a light microscope using a 40× objective.

### Western blot analysis

Western blotting was performed as described previously[1, 20]. Briefly, the cellular proteins were fractionated by 10% SDS-PAGE gel and electroblotted onto Hybond ECL membranes (Amersham Pharmacia, NJ). After being blocked with nonfat milk, the membranes were blotted overnight at 4°C with following primary antibody [anti-TLR9, anti-phospho-p38, anti-p38, anti-phospho-Akt, anti-Akt, anti-cleaved-caspase-3, anti-caspase-3, anti-PARP, anti-Bcl-2, anti-Bax, anti-phospho-GSK-3β, anti- GSK-3β, anti-GAPDH (Cell Signaling Technology, Beverly, MA)][20]. Next day, after incubation with HRP-conjugated secondary antibodies (Cell Signaling Technology, Inc.), membranes were then developed with the Super Signal West Dura Extended Duration substrate (Pierce Biotechnology, Rockford, IL). The bands were quantified by densitometry using a Bio-Image Analysis System (Bio-Rad).

### Enzyme linked immunosorbent assay (ELISA) for cytokines

Equal amounts of peritoneal macrophages (5×10^5^ cells/ mL) were planted in 96-well plates. The supernatants were harvested after 24 h of incubation. The concentration of cytokines in the supernatants was detected by ELISA kits (R&D Systems, Minneapolis, MN) according to our previous studies [5].

### Statistical analysis

Data were expressed as mean ± S.E.M. Statistical analysis were performed using one-way analysis of variance (ANOVA) followed by Bonferroni tests to examine whether differences among groups existed. A P value < 0.05 was accepted as significant.

## Results

### TLR9 is required for chronic stress-induced macrophages accumulation

**Fig 1:**
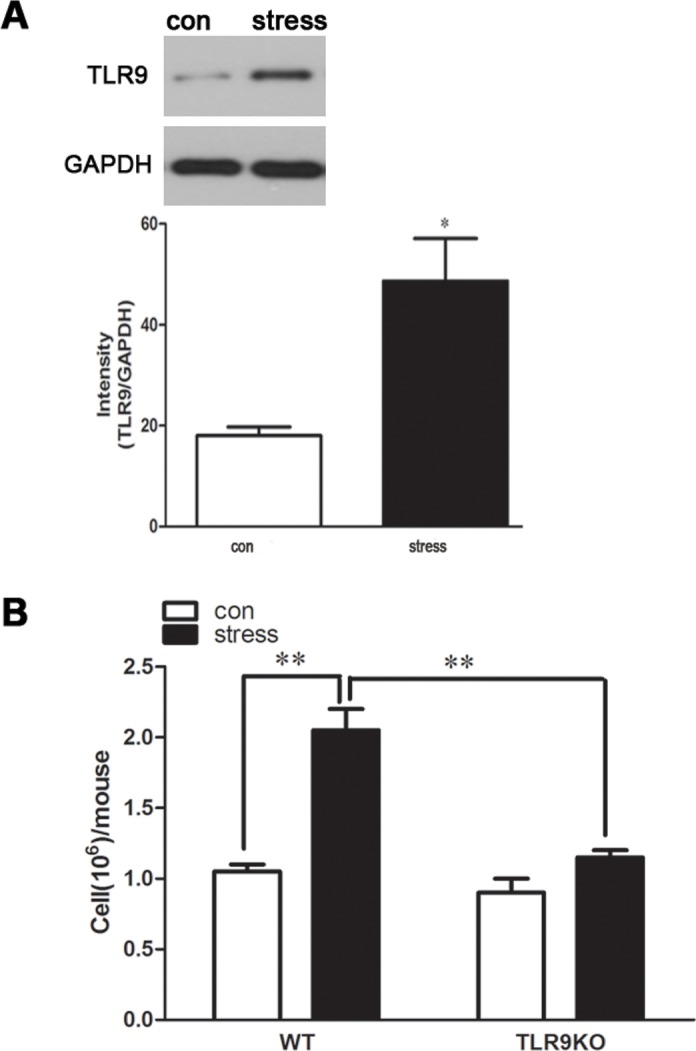
A deficiency of TLR9 blocks chronic stress-induced accumulation of macrophages in peritoneal cavity TLR9 knockout mice or wild type BALB/c mice aged 6 to 8 weeks were subjected to a 12 h physical restraint daily. After 2 d stress, mice were sacrificed by cervical dislocation, and the peritoneal macrophages were harvested and the counts were performed. For TLR9 protein expression evaluating, the macrophages were harvested and cultured for 24 hours. The expression of TLR9 was analyzed by Western blot. Means and SEs were calculated from 7 mice per group. ^*^ *p < 0*.*05*, ^**^ *p< 0*.*01* compared with indicated groups.

Recent evidence showed that TLR9 is largely expressed on macrophages, however, the exact role of TLR9 in modulating macrophage function is not known yet [24]. Data suggests that chronic stress increases TLR9 expression in peritoneal macrophage (Fig 1A). We therefore asked whether TLR9 is involved in stress-mediated changes of macrophage function. Since macrophages in peritoneal cavity of chronic stress-induced mice, irrespective of their location, can significantly contribute to inflammation and immune response by producing cytokines and free oxygen radicals [25] [26], it is important to assess the total number of macrophages accumulated in peritoneal cavity. Therefore, we decided to examine the pattern of total macrophages increase after stress treatment in the peritoneal cavity of TLR9 knockout and wild type mice. We observed a robust accumulation of macrophages in peritoneal cavity 2 days after stress challenge, representing a > 2-fold increase over baseline number of cells; no significant change in number of peritoneal macrophages was observed after stress challenge compared with that in control group in TLR 9 knockout mice (Fig 1B). Therefore, TLR9 knockout mice lose their sensitivity to chronic stress-induced accumulation of macrophages, supporting a critical role of TLR9 in stress-induced immune response.

### Macrophages from TLR9 knockout mice display impaired changes of chronic stress-induced cytokine levels

**Fig 2:**
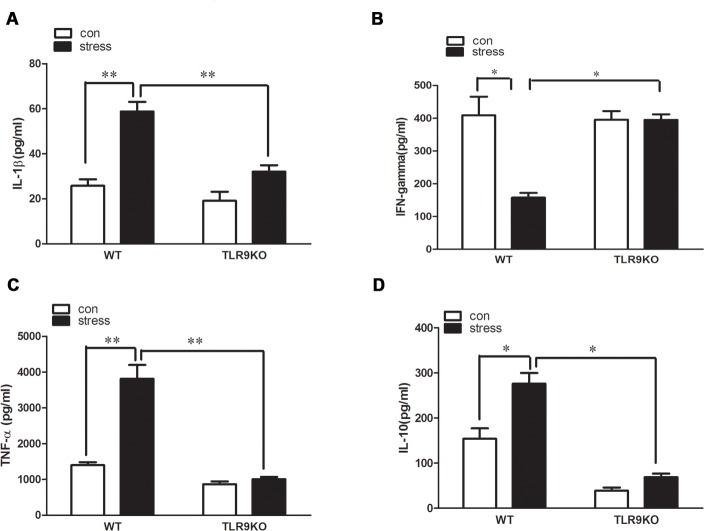
A deficiency of TLR9 decreases chronic stress-induced changes of pro-inflammatory cytokine levels by macrophages TLR9 knockout mice or wild type BALB/c mice aged 6 to 8 weeks were subjected to a 12 h physical restraint daily. After 2 d stress, mice were sacrificed by cervical dislocation, and the macrophages were harvested, purified and cultured (5 × 10^5^ cells/well) on culture plates for 24 hours. IL-1β, TNF-α, IL-10 and IFN-γ levels were measured in supernatants of macrophages by ELISA kit. Means and SEs were calculated from 7 mice per group. ^*^ *p < 0*.*05*, ^**^ *p< 0*.*01* compared with indicated groups.

In response to a large range of stimulation, macrophages secrete powerful biological substances, such as TNF-αand interleukins. This secretion results in inflammation. To investigate whether the diminished accumulation observed in the TLR9 knockout mice was secondary to altered macrophage function, we have detected generation of major pro-inflammatory cytokines by macrophages. Wild type and TLR9 knockout mice were subjected to stress as described previously, peritoneal macrophages were harvested and cultured for 24 hours. Our data showed that IL-1β, TNF-α, IL-10 were significantly overproduced in supernatants of macrophages of stressed wild type mice, increasing by 2.2-, 3.1- and 1.8-fold, compared to that of control wild type mice, respectively. However, IL-1β, TNF-α, IL-10 levels of stressed TLR9 knockout mice displayed no distinctive change compared to control TLR9 knockout mice and (Fig 2A, 2C and 2D). Chronic stress significantly inhibited IFN-γ production in supernatant of macrophages from stressed wild type mice by 2.5-fold than that from control wild type mice, but no change in IFN-γ expression level in supernatant of macrophages was observed after chronic stress challenge in TLR9 knockout mice (Fig 2B).

### TLR9 deficiency blocks chronic stress-induced changes of pro-inflammatory cytokines in serum

**Fig 3:**
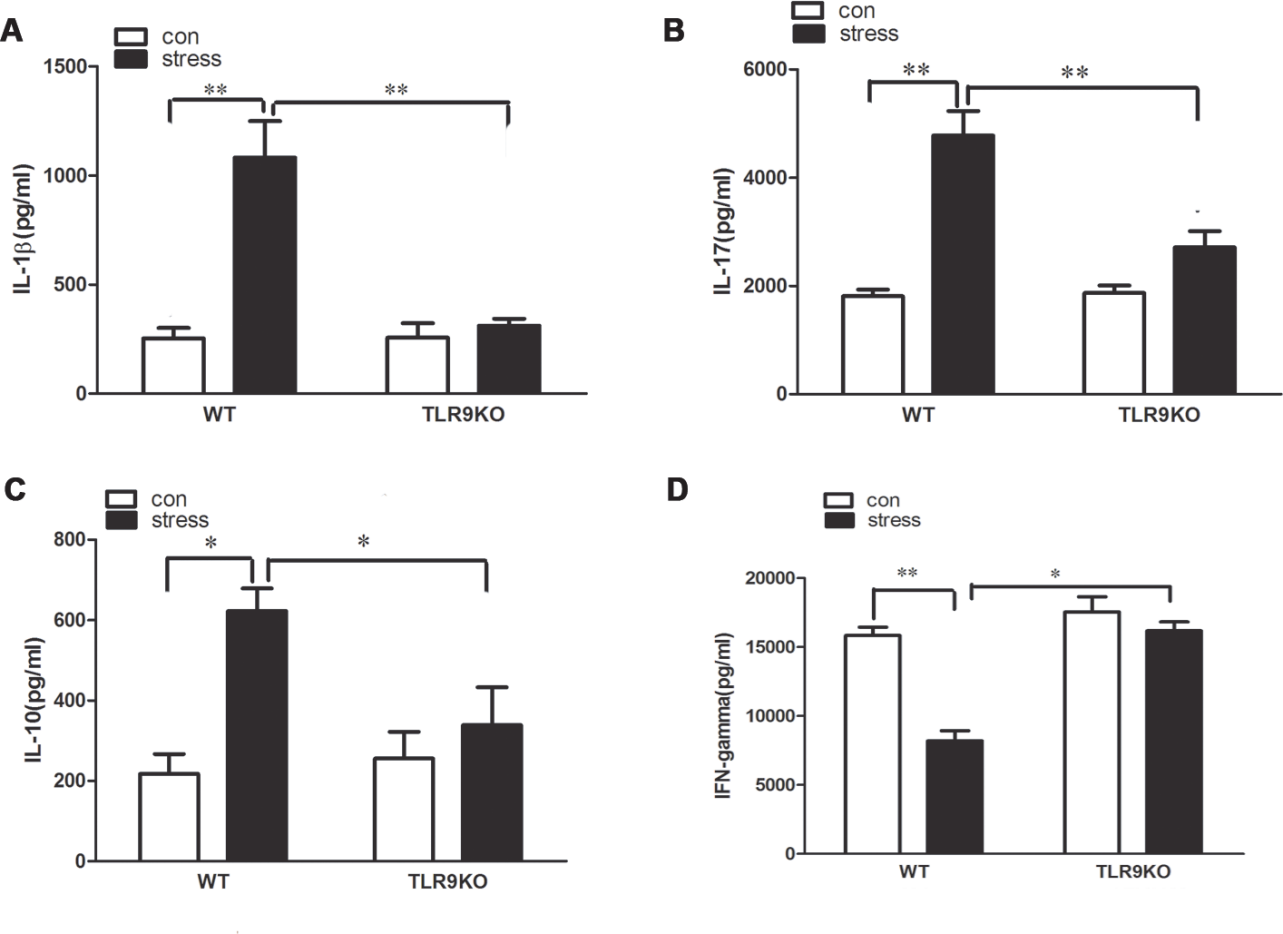
A deficiency of TLR9 suppressed change of cytokine levels caused by chronic stress. TLR9 knockout mice or wild type BALB/c mice aged 6 to 8 weeks were subjected to a 12 h physical restraint daily. After 2 d stress, mice were sacrificed by cervical dislocation, and the serum were harvested and the levels of IL-1β, IL-10, IL-17 and IFN-γ in serum were examined by ELISA kit. Means and SEs were calculated from 7 mice per group. ^*^ *p < 0*.*05*, ^**^ *p< 0*.*01* compared with indicated groups.

It is known that excessive production of plasma proinflammatory cytokines in response to chronic stress can promote the development of immune suppression. We next assessed the expression of IL-1β, IL-10, IL-17 and IFN-γ in the serum of wild type and TLR9 knockout mice challenged with chronic stress. Our data showed that expression level of IL-1β, IL-10, IL-17 in the serum of stressed wild type mice increased by 3.3-, 2.9- and 2.8-fold, compared to that of control wild type mice, respectively. However, the expression level of IL-1β, IL-10, IL-17 did not differ between the control TLR9 knockout mice and stressed TLR9 knockout mice (Fig 3A, 3B and 3C). Chronic stress significantly inhibited IFN-γ production in the serum from stressed wild type mice by 2.1-fold than serum from control wild type mice, but chronic stress failed to induce the change in TLR9 kncokout mice (Fig 3D).

### TLR9 deficiency blocks chronic stress-induced macrophage apoptosis

**Fig 4:**
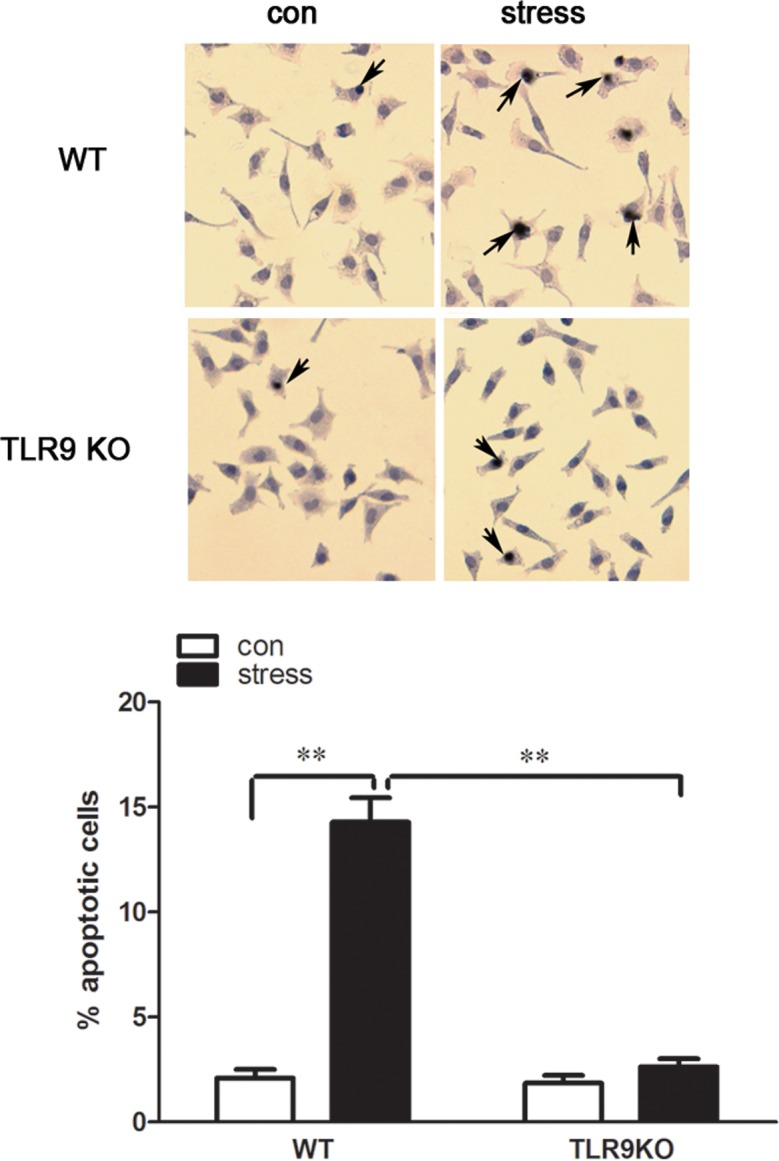
A deficiency of TLR9 is resistant to stress-induced macrophage apoptosis TLR9 knockout mice or wild type BALB/c mice aged 6 to 8 weeks were subjected to a 12 h physical restraint daily. After 2 d stress, mice were sacrificed by cervical dislocation, and the macrophages were harvested, purified and cultured (5 × 10^5^ cells/well) on culture plates for 24 hours. Apoptotic cells (dark brown color cells) were determined by TUNEL assay. Photographs of representative TUNEL-stained cells are shown at the top. Magnification 200×. The bar graph shows the percentage of apoptotic cells. Means and SEs were calculated from 7 mice per group. ^*^ *p < 0*.*05*, ^**^ *p< 0*.*01* compared with indicated groups.

Our recent study showed that chronic stress induces lympocyte apoptosis [2]. We also reported that chronic stress promotes cell apoptosis through TLR9 [12]. To determine whether TLR9 is associated with stress-induced macrophages apoptosis, wild type and TLR9 knockout mice were subjected to stress as described previously, peritoneal macrophages were then harvested and cultured for 24 hours and TUNEL assay was performed to detect cell apoptosis. We found that a large amount of wild type macrophages were undergoing apoptosis after restraint stress, whereas only a few apoptotic cells were detected in the TLR9 deficient macrophages following stress challenge (Fig 4). Therefore, stress-induced macrophage apoptosis requires TLR9.

### TLR9 deficiency attenuates stress-induced activation of caspase-3 and PARP and alteration of Bcl-2/Bax ratio

**Fig 5:**
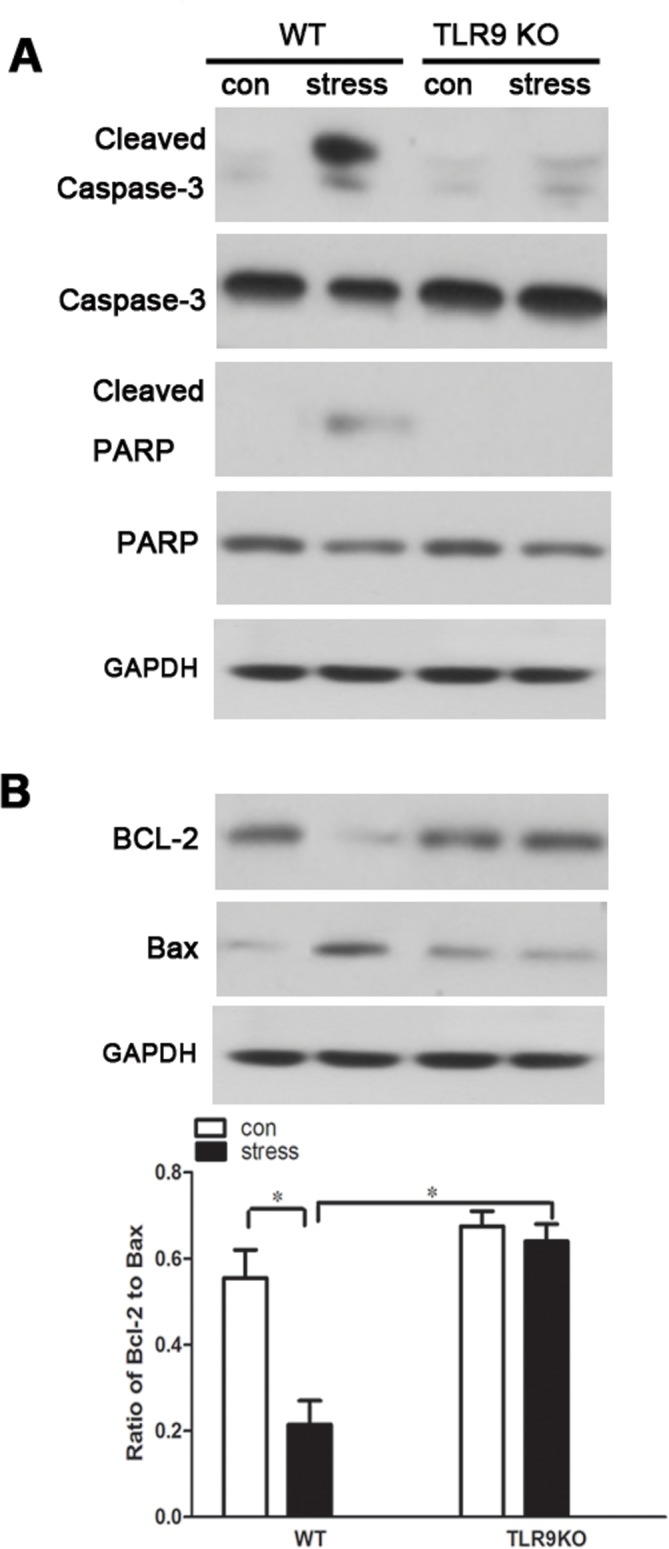
TLR9 deficency inhibits stress-induced change in caspase-3 and PARP activation and ratio of Bcl-2/Bax TLR9 knockout mice or wild type BALB/c mice aged 6 to 8 weeks were subjected to a 12 h physical restraint daily. After 2 d stress, mice were sacrificed by cervical dislocation, and the macrophages were harvested, purified and cultured (5 × 10^5^ cells/well) on culture plates for 24 hours. The expression of cleaved caspase-3 and cleaved PARP (A), and Bcl-2/Bax (B) was analyzed by Western blot. Means and SEs were calculated from 7 mice per group. ^*^ *p < 0*.*05*, ^**^ *p< 0*.*01* compared with indicated groups.

The levels of major apoptosis-related proteins were detected to further assess the mechanisms underlying cellular changes observed in mice after stress challenge. We found that the levels of cleaved caspase-3 and cleaved PARP, two well-known characteristics of apoptosis, were remarkably increased in macrophages of wild type mice following stress treatment, whereas the increases of cleaved caspase-3 and cleaved PARP were attenuated markedly in TLR9 deficient macrophages (Fig 5A). As protein expressions of Bcl-2 and Bax are involved in the chronic stress-induced apoptotic pathway [27], we examined the ratio of Bcl-2 and Bax in macrophages to elucidate the mechanism of stress-induced apoptosis. Stress challenge markedly decreased the ratio of Bcl-2/Bax in wild type macrophages; moreover, the expressions of Bcl-2 and Bax in the macrophages were not altered in TLR9 deficient mice. Our data suggested that Bcl-2 family participate in TLR9-mediated macrophage signaling after stress treatment (Fig 5B).

### TLR9 deficiency blocks chronic stress-induced changes of apoptosis related pathways

**Fig 6:**
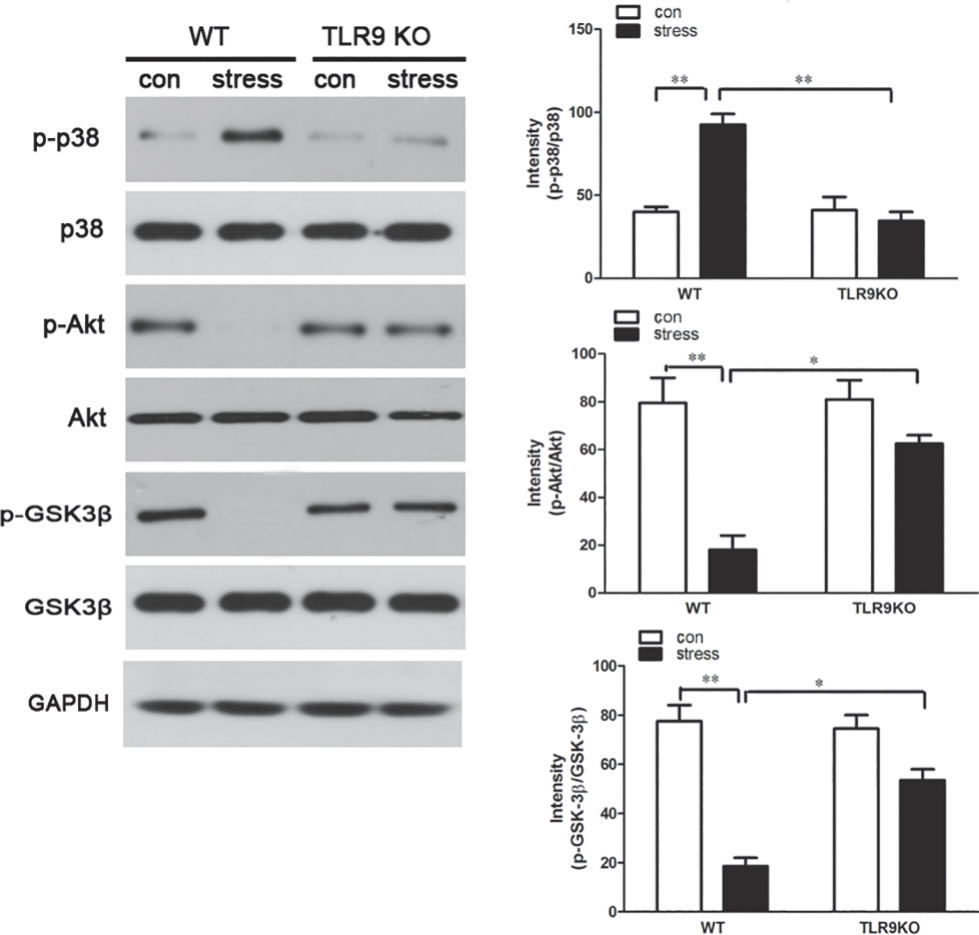
TLR9 deficiency attenuates chronic stress-induced changes of apoptosis related pathways TLR9 knockout mice or wild type BALB/c mice aged 6 to 8 weeks were subjected to a 12 h physical restraint daily. After 2 d stress, mice were sacrificed by cervical dislocation, and the macrophages were harvested, purified and cultured (5 × 10^5^ cells/well) on culture plates for 24 hours. The expression of total and phospho-p38, total and phospho-Akt, total and phospho-GSK-3β were analyzed by Western blot. Means and SEs were calculated from 7 mice per group. ^*^ *p < 0*.*05*, ^**^ *p< 0*.*01* compared with indicated groups.

Accumulating evidence indicates that p38 MAPK participates as a modulator in Bcl-2/Bax-mediated apoptosis in neuroblastoma cells [28] [29]. It also has been demonstrated that activated Akt alters the ratio of Bcl-2 and Bax and exhibits an anti-apoptotic role in various cells [30]. Additionally, recent studies have revealed cross-talk between TLR signaling and the Akt/GSK-3β or p38 MAPK signaling pathway [13] [31]. To examine whether chronic stress activates p38 MAPK and Akt/GSK-3β signaling in TLR9-mediated signaling, the levels of phosphorylated p38 (phospho-p38), phospho-Akt and phospho-GSK-3β in macrophages following stress treatment were examined by western blot analysis. The results of the present study confirmed that chronic stress promoted p38 phosphorylation in wild type macrophage. Moreover, stress-induced p38 MAPK activation was reversed in TLR9 knockout macrophages suggesting that stress markedly increases the level of phospho-p38 through TLR9 (Fig 6). We also found that the increasing levels of phospho-Akt and phospho-GSK-3β were significantly abolished by chronic stress in wild type macrophages but not in TLR9 deficient macrophages, demonstrating that chronic stress decreases the activation of phospho-Akt/phospho-GSK-3β signaling in a TLR9-dependent manner (Fig 6).

## Discussion

The knowledge on pattern recognition receptors (PRRs) recognition and activation of an efficient immune response against chronic stress has progressively increased, mainly in regards to TLR2, TLR4 [5, 27]. Several lines of evidence suggest that TLR9 seems to participate in chronic stress-mediated immune suppression, however, the involvement of TLR9 in functional change of macrophages following chronic stress treatment has not yet been addressed. Our data presented herein clearly revealed that chronic stress might act through TLR9 to generate macrophage inflammation and apoptosis, further supporting that TLR9 plays a role in immune response.

In response to multiple waves of pathogenic stimuli, inflammatory mediators including TNF-α, IFN-γ, IL-1β, IL-6 and IL-10 may be liberated by macrophages. These molecules with diverse physiological effects might play critical roles in the recruitment and apoptosis of macrophages. Indeed, in the present study, we showed that chronic stress induced series inflammatory response characterized by recruitment of macrophages into the peritoneum; generation of pro-inflammatory mediators from macrophages, such as IL-1β and TNF-α and induction of apoptosis. Interestingly, TLR9 deficiency markedly diminished these inflammatory responses induced by chronic stress.

Our previous study indicates that chronic stress causes an imbalance in the Th1 and Th2 responses [32]. Increase of IL-1βand TNF-αsecretion in blood and brain turns out to be a common feature of diverse models of stress [33], whereas the decrease of other cytokines like IFN-γ and IL-10 seems to be more controversial [34–36]. IL-17, an important cytokine produced by Th17 cells, is able to indirectly induce the recruitment of macrophages and neutrophils during inflammation[37]. In fact we showed in this study that chronic stress could induce a dramatic increase of IL-1β, IL-10 and IL-17 production and a significant decrease of IFN-γsynthesis.In contrast, the production of these cytokines was almost unaffected in TLR9 deficient mice under chronic stress influence. These results corroborate a previous report showing that malaria in TLR9 knockout mice significantly diminishes changes of Th1 and Th2 cytokines as compared to control wild type mice [38], indicating that chronic stress leads to immune suppression in a TLR9-dependent manner.

We next attempted to investigate the mechanistic pathway by which TLR9 modulates immune responses. During the process of apoptosis several targets have been identified as characteristic of cell death, including the potential decreases in mitochondrial membrane [25]. Apoptotic stimulation causes the change in mitochondrial membrane potential and the release of cytochrome C into the cytoplasm, activating caspase-9, triggering activation of other caspase members, including caspase-7 and caspase-3, to initiate a caspase cascade, which leads to apoptosis [39, 40]. Furthermore, PARP acts as the main cleavage targets of caspase-3 [41]. For these reason, we detected the expression of cleaved-caspase-3 and cleaved-PARP. In our current study, we demonstrated that chronic stress dramatically upregulated cleavage of caspase-3 and PARP in wild type macrophages. This agrees with our previous report on chronic stress-induced apoptosis accompanied by caspase-3 activation in splenocytes [27]. We also found that the elevated activation of caspase-3 as well as PAPR was significantly blocked to almost control level in TLR9 deficient macrophages. In addition, apoptosis is commonly associated with an imbalance between pro- and anti-apoptotic members of the Bcl-2 family. In the current study, the ratio of Bcl-2/Bax was markedly reduced in chronic stress-induced macrophage, indicating that cells were undergoing apoptosis, however, TLR9 deficiency elevated the Bcl-2/Bax ratio remarkably in macrophage, further confirming that TLR9-deficient macrophage was resistant to chronic stress-induced apoptosis.

Accumulating evidence suggests that p38 MAPK and Akt signaling pathways acts to regulate the cell cycle progression and proliferation [42] [43]. The data of current study indicated that the p38 signaling pathway was significantly activated by chronic stress treatment in wild type macrophages, indicating that p38 was involved in stress-induced macrophage apoptosis. In contract, stress-induced p38 activation was suppressed in TLR9 knockout macrophages, suggesting that stress promoted p38 phosphorylation through TLR9. Consistently, a recent study showed that activation of p38 MAPK signaling pathway by morphine induced apoptosis in a TLR9 dependent manner [31]. As recent evidence implicates, there is a cross-talk between TLR signaling and the Akt/GSK-3β signaling pathway. In previous studies we documented that TLR2 was required for chronic stress-induced apoptosis via PI3K/Akt/GSK-3β signaling cascade in lymphocytes [9]. Additionally, the PI3K/Akt signaling cascade may participate in TLR4-mediated immune responses as an endogenous negative feedback regulator [5]. Considering that Akt/GSK-3β has an important role on immune cells activation in a TLR dependent manner, we then addressed the question of whether this signaling pathway was associated with chronic stress-induced macrophage apoptosis. As expected, no phosphorylation was detected in macrophages when wild type mice were subjected to chronic stress. In contract, phosphorylation of Akt and GSK-3β was restored to the normal level in TLR9 deficient macrophages, suggesting that in TLR9 deficient mice, the higher level of phosphor-Akt and phosphor-GSK-3β prevents macrophages from stress-induced inflammation and apoptosis. Collectively, our data implicates that TLR9 participates in chronic stress-induced immune response via mediating apoptosis-related signaling pathways and proteins.

In summary, our data demonstrated that the chronic stress plays immunosuppressive function partially by inducing macrophage responses and was characterized by a vigorous TLR 9-mediated accumulation of macrophages and release of cytokine, resulting in alteration of macrophage cell signaling and immunosuppression. Theses cytokines might further participate in the apoptosis of macrophages. Bcl-2 family and caspase-3; p38 MAPK and Akt/GSK-3β signaling take part in TLR9-mediated chronic stress-induced apoptosis in macrophage.
